# Acute positional vertigo in the emergency department—peripheral vs. central positional nystagmus

**DOI:** 10.3389/fneur.2023.1266778

**Published:** 2023-10-05

**Authors:** Nehzat Koohi, Amanda J. Male, Diego Kaski

**Affiliations:** Department of Clinical and Movement Neurosciences, The UCL Queen Square Institute of Neurology, University College London, London, United Kingdom

**Keywords:** central positional nystagmus, acute vertigo, BPPV, vestibular migraine, emergency department

## Abstract

**Introduction:**

Benign paroxysmal positional vertigo (BPPV) is the most common cause of positional vertigo. However, positional vertigo can also be due to diseases affecting the central vestibular pathways, such as vestibular migraine. Accurate and timely diagnosis enables effective triage and management.

**Objectives:**

To evaluate diagnoses made by emergency clinicians compared to acute vertigo specialists, in patients presenting to an emergency department (ED) with positional vertigo.

**Methods:**

Following routine ED care, patients with a primary complaint of dizziness, vertigo, light-headedness or unsteadiness, underwent detailed neuro-otological assessment by acute vertigo specialists. Demographics and final diagnoses were recorded and analyzed.

**Results:**

Of 71 consented patients (21−91 years; mean 56 years, ±16.7 years, 40 females), ED identified 13 with a peripheral cause of positional vertigo (mean 48.85 years, ±16.19, 8 females). Central positional nystagmus was not noted in any of the patients with positional vertigo seen by the ED clinicians. Acute vertigo specialists diagnosed nine patients with BPPV (age range 50-88 years, mean 66 years, ±12.22, 5 females), and six with central positional nystagmus (age range 23−59 years, mean 41.67 years, ±15.78, 6 females).

**Conclusion:**

Positional vertigo should be assessed with positional maneuvers such as Dix-Hallpike and Roll tests in the ED to identify peripheral and central nystagmus features. Central causes are more common in younger females, with the presence of vomiting, and/or a background of motion sensitivity.

## Introduction

Positional vertigo is a symptom that is triggered by the act of moving the head to a new position (1). It can be due to peripheral (inner ear) or central causes, and have either a persistent or paroxysmal presentation (2). Benign paroxysmal positional vertigo (BPPV) is considered the most common peripheral cause for positional vertigo (3) and is due to otoconial debris becoming displaced from the maculae into a semicircular canal. It affects all age groups, but peaks in the sixth decade (4). It is characterized by brief vertigo and triggered by changes in head position against gravity. Conversely, central positional nystagmus (CPN) is attributed to disease affecting the central nervous system and is an important differential diagnosis to BPPV. CPN has been reported in a range of neurological disorders (5), including multiple sclerosis (6), cerebellar disease (7), and cerebellar stroke (8), with the commonest central cause being vestibular migraine (VM) particularly during an attack (9, 10).

The pathophysiology of CPN is poorly understood but it is thought be due to an impaired integration of semicircular canals and central vestibular pathways in the cerebellum and brainstem and consequently providing a poor estimation of head tilt and/or eye position coordinates (9). Features of CPN include: (a) nystagmus that is atypical for BPPV is observed in the positional maneuvers, (b) the nystagmus fails to resolve with treatment, (c) additional neurological or ocular motor symptoms signs may be present (5, 9). This highlights the importance of completing positional tests and an oculomotor assessment to differentiate central from peripheral causes (2, 11, 12), preferably with nystagmus documentation using video oculography.

Misclassification of the signs in patients with acute positional vertigo presenting to the ED has the potential to lead to misdiagnosis and mismanagement. Identifying the clinical presentations and signs in patients with positional vertigo, particularly in those with atypical positional nystagmus can help early and accurate identification of peripheral or central causes of acute positional vertigo. This will ensure appropriate repositioning maneuvers are completed when the cause is peripheral (BPPV can be quickly diagnosed and managed in the ED), and effective timely triage and management when central.

The aim of this article is to define the proportion of adults attending a single tertiary United Kingdom emergency department with positional dizziness or vertigo and identify the prevalence of BPPV versus central positional nystagmus, including vestibular migraine. We present the diagnoses made by both the ED clinicians and acute vertigo specialists. Furthermore, we describe the CPN features and clinical characteristics that aid the identification of central versus peripheral causes of positional vertigo.

## Method

A prospective observational descriptive study was conducted on patients with a primary complaint of ‘dizziness’, ‘unsteadiness’, ‘light-headedness’, or ‘vertigo’ attending the ED of University College London Hospitals in the United Kingdom between January 2022 and December 2022. We included adult (18-90 yrs) patients who were able to consent. Patients with pre-existing dizziness and vertigo before attending the ED were not included. Ethical approval was obtained from the United Kingdom Northwest—Greater Manchester South Research Ethics Committee (approval No. 21/ NW/0015). This is a sub-study of a larger research project, for which a manuscript is under preparation.

Initial ED assessment was performed by a resident neurologist or emergency physician, under direct consultant supervision. Such assessment typically includes a full clinical history and general medical and neurological assessment, but may not always include HINTS-plus or positional maneuvers. Acute vertigo specialists were a neurologist (DK) with expertise in neuro-otology, and a Clinical Scientist in Neuro-audiology (NK) with expertise in audiovestibular science, with a combined experience of over 20 years in the field. All consented patients underwent the ED routine care and were later seen by the acute vertigo specialists. Neuro-otological assessments were performed by the specialist no later than 5 days of symptom onset. To exclude the presence of any associated signs, clinical examination that included spontaneous nystagmus, saccades, pursuit, bedside and video head impulse test, and Dix-Hallpike and Roll positional tests, with and without videonystagmography goggles (ICS Impulse; Natus, Denmark) were performed. All patients underwent computerized magnetic resonance imaging of the brain with diffusion angiography, T1, T2, and fluid-attenuated inversion recovery no later than 72 h of admission as part of the research study protocol, to systematically screen for central causes.

Continuous data is reported as means and standard deviations, categorical data presented as counts and proportions, and with *p*-values and z-scores calculated.

## Results

A total of 71 adults presented to the ED reporting ‘dizziness’, ‘unsteadiness’, ‘light-headedness’, or ‘vertigo’ were consented and able to be seen by the acute vertigo specialist team. Age range for the whole group was 21-91 years (mean 56 years, ±16.76 years), with more females (40 females to 31 males).

**Figure 1:**
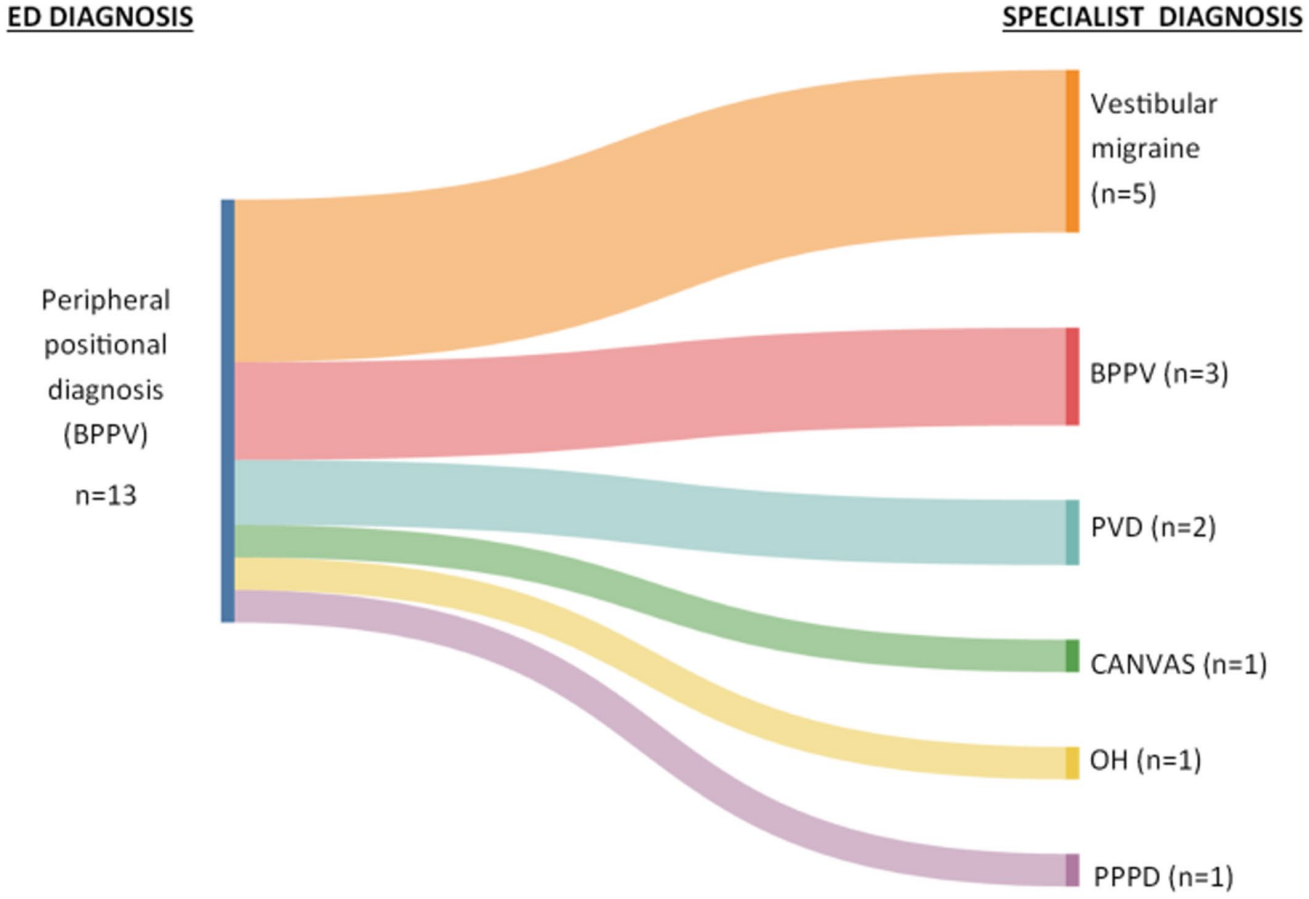
Sankey diagram showing ED diagnoses and final diagnoses made by specialists. BPPV, benign paroxysmal positional vertigo; PVD, peripheral vestibular dysfunction; CANVAS, cerebellar ataxia, neuropathy, vestibular areflexia syndrome; OH, orthostatic hypotension; PPPD, persistent postural perceptual dizziness.

ED diagnosed 13 patients (age range 26 to 80 years, mean 48.85 years, ±16.19, 8 females) with a peripheral positional cause (i.e., BPPV), with no documentation of side or affected canal. CPN was not noted in any of the patients seen by the ED clinicians. Figure 1 shows those diagnosed by ED with a peripheral positional cause and the final diagnoses made by the acute vertigo specialists.

ED clinicians completed diagnostic positional maneuvers in 20% (*n* = 14) of the whole cohort, and specialists in 99% (*n* = 70). Of the 13 patients (18% of total cohort) that ED clinicians diagnosed as BPPV, the Dix-Hallpike test was completed only in seven. Of these seven, five were reported as having a positive Dix-Hallpike (of whom three patients received treatment repositioning manoeuvrers), and two were given a diagnosis of ‘resolved’ BPPV (the Dix-Hallpike was reported as negative in these two patients). Six patients (out of 13) were diagnosed with BPPV without positional maneuvers being performed on them.

Acute vertigo specialists diagnosed nine patients with a peripheral cause of positional vertigo, four with posterior canal BPPV, two with horizontal canal BPPV, and three resolved BPPV after ED performed repositioning maneuvers (age range 50 to 88 years, mean 66 years, ±12.22, 5 females), and six with a central cause of positional vertigo with apparent CPN (age range 23 to 59 years, mean 41.67 years, ±15.78, 6 females). The difference in ages was statistically significant between peripheral and central groups (older in the BPPV group, *p* = 0.005), and females between diagnoses (more females in the CPN group, *p* = 0.024).

**Figure 2:**
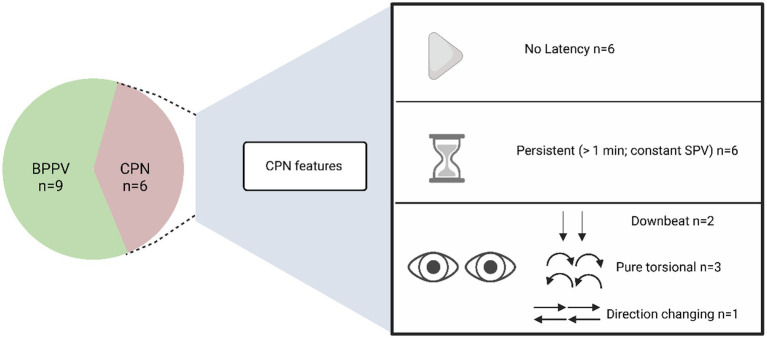
Proportion of peripheral and central causes of positional vertigo made by acute vertigo specialists, and features of CPN. CPN, central positional nystagmus; BPPV, benign paroxysmal positional vertigo; SPV, slow phase velocity.

Acute vertigo specialists detected CPN in six patients with acute positional vertigo, all of whom had a clinical diagnosis of vestibular migraine. The nystagmus features are summarized in Figure 2.

The MRI did not show any structural lesions in any of the patients with positional vertigo assessed by the acute specialists. Other vestibular and oculomotor examinations including spontaneous nystagmus, saccades, pursuit, and head impulse test were unremarkable (Table 1).

**Table 1:** Characteristics (the presenting complaint, triggers, duration, associated symptoms and vascular risk factors) of the patients diagnosed by specialists with either peripheral (BPPV) or central positional (VM) diagnoses.

	Specialist peripheral diagnosis *n* = 9 (%)	Specialist central diagnosis *n* = 6 (%)	Value of *p*	z-score
Presenting complaint				
Dizziness	1 (11)	2 (33)	NS	
Vertigo	9 (100)	6 (100)	NS	
Light-headedness	2 (22)	1 (17)	NS	
Oscillopsia	3 (33)	1 (17)	NS	
Imbalance	2 (22)	3 (50)	NS	
Unsteadiness	1 (11)	3 (50)	NS	
Other	0	0		
Symptom duration				
Seconds	2 (22)	0	NS	
Minutes	7 (78)	3 (50)	NS	
Hours	0	1 (17)	NS	
Days	0	2 (33)	NS	
Triggers				
Head motion	6 (67)	2 (33)	NS	
Head position	6 (67)	4 (67)	NS	
Orthostatic change	1 (11)	0	NS	
Exertion	0	0		
Head turning	5 (55)	3 (50)	NS	
Complex visual environments	0	2 (33)	NS	
Passive self-motion	1 (11)	1 (17)	NS	
Walking on uneven surfaces (or in the dark)	0	0		
No triggers	0	1 (17)	NS	
Other	1 (11)	1 (17)	NS	
Associated symptoms				
Nausea	7 (78)	4 (67)	NS	
Vomiting	**0**	**4 (67)**	**0.004**	**−2.9**
Deafness	0	0		
Tinnitus	0	1 (17)	NS	
Aural Fullness	0	0		
Headaches	4 (44)	4 (67)	NS	
Neurological Symptoms	0	0		
Anxiety	2 (22)	2 (33)	NS	
Other	0	0		
Motion sensitivity				
Yes	**0**	**6 (100)**	**0.0003**	***−3.8***
No	**9 (100)**	**0**	**0.0003**	**3.8**
Previous history of headaches				
Yes	2 (22)	3 (50)	NS	
No	7 (78)	3 (50)	NS	

Weighted comparisons are calculated and presented as *p* = values and *z* = scores. NS, not significant. Bold font indicates statistical significance.

## Discussion

This study investigated patients presenting to an ED with acute dizziness or vertigo, focusing on positional nystagmus. The diagnoses made by the ED clinicians were compared to the diagnoses made by the acute vertigo specialists.

ED diagnosed peripheral causes of positional vertigo (i.e., BPPV) in 18% of the total cohort, with no patients diagnosed with central causes of acute positional vertigo. While this proportion of patients diagnosed with BPPV in ED in this study is comparable to that previously reported (13), the ED clinicians completed positional maneuvers in just seven of the 13 patients they diagnosed with BPPV, with some maneuvers reported as negative despite a final diagnosis of BPPV. In addition, only three of those that ED diagnosed with BPPV went on to have confirmed BPPV diagnosis by the acute vertigo specialists (Figure 1). These findings highlight the importance of completing positional maneuvers in an acute setting to guide accurate diagnosis and management (14).

Of 71 consented patients, acute vertigo specialists identified six patients with CPN, confirmed and documented by video-nystagmography-oculography during the positional tests. When completing positional tests for BPPV, the nystagmus triggered had typical features, namely a delay (latency) to nystagmus onset, a torsional-vertical component toward the under most ear, and a crescendo-decrescendo pattern that resolves within 20 s (15). The features of CPN were quite different to those of peripheral nystagmus (Figure 2). This highlights the importance of accurate interpretation of the nystagmus seen in positional tests in an acute setting. Acknowledging central positional nystagmus interpretation can be challenging to the untrained ED clinician, recognizing that it differs from peripheral nystagmus in all three characteristics reported in the seminal work by Dix and Hallpike (15) (onset, duration and direction), would enable non peripheral nystagmus to be identified and suitable onward referrals made. Certain atypical, and rarer forms of BPPV can present with nystagmus that would be more typical for a central cause (e.g., positional downbeat nystagmus in anterior canal BPPV, or nystagmus that does not fatigue in cupulolithiasis-type posterior canal BPPV). Diagnosing central positional nystagmus remains a clinical challenge due to the overlap in oculomotor characteristics with BPPV and is largely based on atypical features for BPPV rather than its own specific characteristics (5).

Although we acknowledge the small sample size of this patient group with acute positional vertigo, salient clinical messages are apparent: (i) the somewhat lower prevalence of acute BPPV in patients with acute positional vertigo in the ED when central causes are also considered, (ii) VM may be the commonest cause of acute central positional vertigo in the ED, (iii) CPN caused by VM is more common than BPPV in younger females (<60 yrs) and those with a background of motion sensitivity, and (iv) vomiting is not a common feature of BPPV so a central cause should be suspected in patients with positional vertigo and vomiting. Larger trials are of course encouraged to validate these findings.

In summary, positional vertigo is more likely to be due to a central cause in younger females, with the presence of vomiting, and/or a background of motion sensitivity. This study reinforces the need for diagnostic positional tests in ED in patients with positional vertigo. This would allow diagnosis to be reached faster, with potential longer-term benefits for patient outcomes, and would also obviate the need for additional investigations and onward referrals.

## Data availability statement

The original contributions presented in the study are included in the article/supplementary material, further inquiries can be directed to the corresponding author.

## Ethics statement

Ethical approval was obtained from the United Kingdom Northwest–Greater Manchester South Research Ethics Committee (approval No. 21/ NW/0015). The studies were conducted in accordance with the local legislation and institutional requirements. The participants provided their written informed consent to participate in this study.

## Author contributions

NK: Formal analysis, Writing – original draft, Writing – review & editing, Visualization, Conceptualization, Data curation, Methodology, Project administration. AM: Formal analysis, Visualization, Writing – original draft, Writing – review & editing, Resources. DK: Formal analysis, Writing – original draft, Writing – review & editing, Conceptualization, Data curation, Funding acquisition, Methodology, Project administration, Supervision, Validation.

## References

[1] Von Brevern M Bertholon P Brandt T Fife T Imai T Nuti D. Benign paroxysmal positional vertigo: Diagnostic criteria. J Vestib Res. (2015) 25:105–17. doi: 10.3233/VES-150553, PMID: 26756126

[2] Choi JY Kim JH Kim HJ Glasauer S Kim JS. Central paroxysmal positional nystagmus: characteristics and possible mechanisms. Neurology. (2015) 84:2238–46. doi: 10.1212/WNL.0000000000001640, PMID: 25957336

[3] von Brevern M Radtke A Lezius F Feldmann M Ziese T Lempert T. Epidemiology of benign paroxysmal positional vertigo: a population based study. J Neurol Neurosurg Psychiatry. (2007) 78:710–5. doi: 10.1136/jnnp.2006.100420, PMID: 17135456 PMC2117684

[4] Baloh RW Honrubia V Jacobson K. Benign positional vertigo: clinical and oculographic features in 240 cases. Neurology. (1987) 37:371–8. doi: 10.1212/wnl.37.3.371, PMID: 3822129

[5] Peña Navarro P Pacheco López S Almeida Ayerve CN Marcos Alonso S Serradilla López JM Santa Cruz Ruiz S. Early diagnosis of central disorders mimicking Horizontal Canal Cupulolithiasis. Brain Sci. (2023) 13:562. doi: 10.3390/brainsci13040562, PMID: 37190527 PMC10137293

[6] Alpini D Caputo D Pugnetti L Giuliano DA Cesarani A. Vertigo and multiple sclerosis: aspects of differential diagnosis. Neurol Sci. (2001) 22:S84–7. doi: 10.1007/s100720100041, PMID: 11794485

[7] Feil K Strobl R Schindler A Krafczyk S Goldschagg N Frenzel C. What Is Behind Cerebellar Vertigo and Dizziness? Cerebellum. (2019) 18:320–32. doi: 10.1007/s12311-018-0992-8, PMID: 30552638

[8] Lee SU Choi JY Kim HJ Park JJ Zee DS Kim JS. Impaired tilt suppression of post-rotatory nystagmus and cross-coupled head-shaking nystagmus in cerebellar lesions: image mapping study. Cerebellum. (2017) 16:95–102. doi: 10.1007/s12311-016-0772-2, PMID: 26969184

[9] Lemos J Strupp M. Central positional nystagmus: an update. J Neurol. (2022) 269:1851–60. doi: 10.1007/s00415-021-10852-8, PMID: 34669008

[10] Macdonald NK Kaski D Saman Y Al-Shaikh Sulaiman A Anwer A Bamiou DE. Central positional nystagmus: a systematic literature review. Front Neurol. (2017) 8:141. doi: 10.3389/fneur.2017.00141, PMID: 28473800 PMC5397512

[11] Choi JY Glasauer S Kim JH Zee DS Kim JS. Characteristics and mechanism of apogeotropic central positional nystagmus. Brain. (2018) 141:762–75. doi: 10.1093/brain/awx381, PMID: 29373699

[12] De Schutter E Adham ZO Kattah JC. Central positional vertigo: A clinical-imaging study. Prog Brain Res. (2019) 249:345–60. doi: 10.1016/bs.pbr.2019.04.022, PMID: 31325993

[13] Cutfield NJ Seemungal BM Millington H Bronstein AM. Diagnosis of acute vertigo in the emergency department. Emerg Med J. (2011) 28:538–9. doi: 10.1136/emj.2010.105874, PMID: 21398692

[14] Rau C Terling L Elkhodair S Kaski D. 92. Acute Vertigo in the emergency department–a retrospective study. Eur J Emerg Med. (2020) 27:e4–5. doi: 10.1097/01, PMID: 33555788

[15] Dix MR Hallpike CS. The pathology, symptomatology and diagnosis of certain common disorders of the vestibular system. Ann Otol Rhinol Laryngol. (1952) 61:987–1016. doi: 10.1177/000348945206100403, PMID: 13008328

